# Correlation between the Glasgow-Blatchford score, shock index, and Forrest classification in patients with peptic ulcer bleeding

**DOI:** 10.3906/sag-1906-154

**Published:** 2020-06-23

**Authors:** Hong YANG, Chen PAN*, Qi LIU, Yan WANG, Zhe LIU, Xian CAO, Jingjing LEI

**Affiliations:** 1 Department of Gastroenterology, The Affiliated Hospital of Guizhou Medical University, Guiyang China; 2 Department of Gastroenterology and Hepatobiliary, The Affiliated Baiyun Hospital of Guizhou Medical University, Guiyang China

**Keywords:** Peptic ulcer hemorrhage, Glasgow-Blatchford score, shock index, endoscopy

## Abstract

**Background/aim:**

To investigate the correlation between the Glasgow-Blatchford score, shock index, and Forrest classification in patients with peptic ulcer bleeding (PUB).

**Materials and methods:**

A total of 955 patients with PUB were assessed using the Glasgow-Blatchford score and shock index, as well as the Forrest classification based on their gastroscopy results. The correlation between the Glasgow-Blatchford score and shock index was determined using scatter plot analysis, and the correlation between the Glasgow-Blatchford score or shock index and Forrest classification was determined using Spearman’s analysis.

**Results:**

Both the Glasgow-Blatchford score and shock index showed the highest values in patients with Forrest class IIa. The Glasgow-Blatchford score was significantly higher than patients with Forrest class Ib/IIc/III (P < 0.05), and the shock index was significantly higher than patients with Forrest class Ib/IIb/III (P < 0.05). A positive correlation was observed between the Glasgow-Blatchford score and shock index, at r = 0.427 (P < 0.001). A negative correlation was observed between the Glasgow-Blatchford score and Forrest classification, at r = –0.111 (P < 0.01), and between the shock index and Forrest classification, at r = –0.138 (P < 0.01).

**Conclusion:**

A moderate correlation was observed between the Glasgow-Blatchford score and shock index in patients with PUB, and the correlation between the Forrest classification and Glasgow-Blatchford score or shock index was relatively low.

## 1. Introduction

Acute nonvariceal upper gastrointestinal bleeding (ANVUGIB) is one of the most severe and life-threatening emergencies, accounting for 80%–90% of acute upper gastrointestinal bleeding cases [1]. The common causes of ANVUGIB include peptic ulcer, upper gastrointestinal tumors, and acute gastric mucosal lesions, and there are other lesions (such as a Mallory-Weiss tear, Dieulafoy lesion, and angiodysplasia) that also cause ANVUGIB. However, at present, peptic ulcer is still the leading cause [2]. In recent years, several risk scores have been used to assess the disease severity, prognosis, and clinical status of patients with ANVUGIB, with the Glasgow-Blatchford score (GBS) [3] being one of the most commonly used in clinical practice. The GBS is based on clinical and laboratory findings without endoscopy results, and was originally designed to predict the need for treatment (including blood transfusion, endoscopy, and surgery. The GBS is currently used to distinguish low-risk patients from high-risk patients, limit the use of medical resources, and reduce hospitalization expenses [4–6].

The shock index (SI) is the ratio of the heart rate to systolic blood pressure, and it is a clinical indicator of hemodynamic status. The SI can be used for the initial monitoring of patients with gastrointestinal bleeding and define treatment and provide an early warning of persistent bleeding or rebleeding after initial therapy [7]. Few studies have investigated the use of the SI in assessing upper gastrointestinal bleeding, and the relationship between the SI and other commonly used risk scores (e.g., the GBS) remains unclear.

Endoscopy is of great importance in the diagnosis and treatment of ANVUGIB [8]. If ANVUGIB patients are definitively diagnosed with peptic ulcer bleeding (PUB) using endoscopy, then the Forrest classification can be determined based on the u2940s of the u2943 base. The Forrest classification is helpful in assessing the risk of rebleeding and can guide proper endoscopic treatment [9–11]. While the GBS, SI, and Forrest classification are often used to assess disease severity, no research to date has investigated the correlation between them. The current study investigated the correlation between the GBS, SI, and Forrest classification in patients with PUB.

## 2. Materials and methods

### 2.1. Study design

This retrospective study considered all of the patients with a diagnosis of PUB who were admitted to the Affiliated Hospital of Guizhou Medical University, Affiliated Baiyun Hospital of Guizhou Medical University, and Cancer Hospital of Guizhou Medical University, between January 2013 and March 2019. All of the patients exhibited hematemesis and/or melena as the main clinical manifestations, and underwent gastroscopy within 24 h of admission, so as to confirm the diagnosis of PUB. The exclusion criteria were as follows: 1) a bleeding site outside of the upper gastrointestinal tract, 2) a malignant tumor, and 3) the absence of the data needed to calculate the GBS. The primary study variables were the GBS, SI, and Forrest classification. This study was conducted in accordance with the declaration of Helsinki and was approved by the Medical Ethical Committee of the Affiliated Hospital of Guizhou Medical University. Written informed consent was obtained from all of the patients prior to their endoscopic procedures. The STROBE guidelines were also followed [12].

### 2.2. Data collection

Primary data were collected from each patient, including their age, sex, medical history (presenting signs or symptoms), comorbidities, use of drugs (aspirin, other antiplatelet drugs, nonsteroidal antiinflammatory drugs (NSAIDs), etc.), smoking, alcohol use, presence of a *Helicobacter pylori* (*H. pylori*) infection (a positive result in a urea breath test or rapid urease test, or histological examination of the gastric mucosa indicating a *H. pylori* infection), history of peptic ulcer or gastrointestinal bleeding, blood pressure, pulse, heart rate, blood urea nitrogen, hemoglobin, endoscopic findings (Forrest classification), need for endoscopic intervention, and treatment. Rebleeding within 7 days after initial therapy (including endoscopic intervention) was also recorded. 

The GBS was calculated for all of the patients based on clinical and laboratory variables (e.g., systolic blood pressure, blood urea nitrogen, hemoglobin, pulse, u2944, etc.) at the time of admission. The SI was calculated according to the heart rate (beats/min) and systolic blood pressure (mmHg) of the patient on admission.

Rebleeding was considered if any of the following events occurred: the reappearance of overt bleeding (new hematemesis or melena), a decrease in systolic blood pressure (≤90 mmHg) or increase in pulse rate (≥110 beats per minute), a decrease in hemoglobin (>20 g/L) within 24 h, or inadequate increase in hemoglobin (<10 g/L) after adequate blood transfusion.

### 2.3. General treatment

The initial general treatment for all of the patients who presented with acute upper gastrointestinal bleeding and awaited endoscopy included monitoring (body temperature, pulse, respiratory rate, blood pressure, urine output, and mental status), bed rest, oxygen inhalation, intravenous rehydration, and initiation of a high-dose intravenous proton-pump inhibitor (PPI), with an intravenous bolus of 80 mg, followed by a continuous infusion of 8 mg/h. Assessments of the bleeding status (u2945s, times, and total amounts of hematemesis and/or melena) and regular reassessment with routine blood tests, including blood urea nitrogen, were performed. Blood transfusion was administered in accordance with the patient’s condition. After endoscopy, patients classified with Forrest Ia to IIb remained on high-dose PPIs via intravenous infusion for 72 h, followed by reduction to a standard dose (2 times each day for 3–5 days) according to their clinical status. The same standard dose was also used for the low-risk patients classified with Forrest IIc and Forrest III. All of these patients who tested positive for *H. pylori *infection were treated with the standard *H. pylori* eradication therapy.

### 2.4. Endoscopy

Endoscopic procedures were performed within 24 h on admission with a CV-260SL or CV-290 gastroscope (Olympus, Tokyo, Japan). After the Forrest classification of the peptic ulcer was defined via endoscopy in all of the patients, endoscopic hemostasis was considered for those patients with high-risk stigmata (Forrest Ia to IIb), while it was not necessary for those with low risk (Forrest IIc and Forrest III). Methods of endoscopic hemostasis included epinephrine injection (epinephrine diluted 1:10,000 in 0.9% saline), electrocoagulation, argon plasma coagulation, hemoclips, etc. The mode of therapy under endoscopy was based on the status of the patient. All of the endoscopic procedures were performed by expert endoscopists who had experience with more than 500 cases of endoscopic hemostasis.

### 2.5. Statistical analysis 

Statistical analysis was performed using SPSS v.20.0 (IBM Corp., Armonk, NY, USA). Quantitative data with a normal distribution were expressed as the mean ± standard deviation (*x̄* ± *s*). A comparison among the Forrest classifications was performed with one-way analysis of variance, and a comparison between the 2 groups was performed using the least significant difference test. Qualitative data were expressed as n (percentage), and the chi-square test and Fisher’s exact test were used for comparisons among the rates, and the chi-square segmentation method was used for comparisons between the 2 groups, with an adjustment of the test level for the rates between the 2 groups to α = 0.0033. The association between GBS and SI was evaluated using Pearson’s correlation analysis, and the association between the GBS/SI and the Forrest classification was evaluated using Spearman’s rank correlation analysis. P < 0.05 was considered statistically significant.

## 3. Results 

### 3.1. Patient characteristics

**Figure 1:**
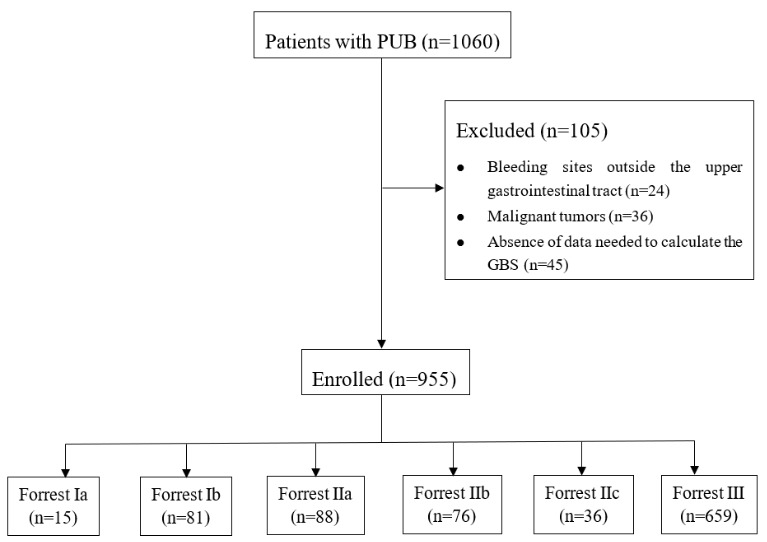
Flowchart showing the flow of patients through the study. Peptic ulcer bleeding (PUB), Glasgow-Blatchford score (GBS).

Between January 2013 and March 2019, a total of 1060 patients with PUB were admitted to the abovementioned institutions. Among those admitted, 24 patients with bleeding sites outside of their upper gastrointestinal tract, 36 with malignant tumors, and 45 without the data required to calculate the GBS were excluded. Finally, a total of 955 patients with PUB were enrolled in this study (Figure 1). The mean age was 57.16 ± 15.12 years, and the group included 701 males (73.40%) and 254 females (26.60%).

Hypertension was present in 286 patients (29.95%), rheumatic disease in 146 (15.29%), diabetes mellitus in 69 (7.23%), and chronic kidney disease in 55 (5.76%). NSAIDs (excluding aspirin) were used in 36.75% of patients, and aspirin was used in 3.46%. Other characteristics, such as smoking, alcohol use, *H. pylori *infection, history of peptic ulcer, or gastrointestinal bleeding, are shown in Table 1. 

**Table 1:** Characteristics of patients with PUB (n = 955).

Patient characteristics	
Age, years, mean ± SD	57.16 ± 15.12
Sex, n (%)	
Male	701 (73.40)
Female	254 (26.60)
Comorbidities, n (%)	
Hypertension	286 (29.95)
Diabetes mellitus	69 (7.23)
Heart failure	27 (2.83)
Cerebrovascular disease	49 (5.13)
Respiratory disease	39 (4.08)
Hepatic disease	44 (4.61)
Chronic kidney disease	55 (5.76)
Rheumatic disease	146 (15.29)
Concomitant drug use, n (%)	
Aspirin	33 (3.46)
Other anti-platelet drugs	19 (1.99)
NSAIDs (excluding aspirin)	351 (36.75)
Warfarin	5 (0.52)
Corticosteroids	24 (2.51)
Smoking, n (%)	462 (48.38)
Alcohol use, n (%)	399 (41.78)
Helicobacter pylori infection, n (%)	
Negative	212 (22.20)
Positive	427 (44.71)
Unknown	316 (33.09)
History of peptic ulcer, n (%)	131 (13.72)
History of gastrointestinal bleeding, n (%)	119 (12.46)

NSAIDs: nonsteroidal antiinflammatory drugs

### 3.2. Endoscopic data 

Endoscopic examination was performed within 24 h of admission in the 955 patients with PUB in the current study. In Figure 1, it can be seen that 15 patients were classified with Forrest Ia, 81 with Forrest Ib, 88 with Forrest IIa, 76 with Forrest IIb, 36 with Forrest IIc, and 659 with Forrest III. Other than the patients with Forrest IIc and Forrest III, all of those with Forrest Ia and most of those with Forrest Ib to IIb underwent endoscopic hemostasis, which included monotherapy and combination therapy (details in Table 2).

**Table 2:** Endoscopic treatment.

Forrest classification	Endoscopic treatment, n (%)	Type of endoscopic treatment
Ia (n = 15)	15 (100.00)	Monotherapy, n = 2 (13.33%)Combination therapy, n = 13 (86.67%)
Ib (n = 81)	75 (92.59)	Monotherapy, n = 20 (26.67%)Combination therapy, n = 55 (73.33%)
IIa (n = 88)	82 (93.18)	Monotherapy, n = 24 (29.27%)Combination therapy, n = 58 (70.73%)
IIb (n = 76)	51 (67.11)	Monotherapy, n = 12 (23.53%)Combination therapy, n = 39 (76.47%)
IIc (n = 36)	0 (0.00)	-
III (n = 659)	0 (0.00)	-

Monotherapy: epinephrine injection, argon plasma coagulation, or hemoclips; combination therapy: combining epinephrine injection with argon plasma coagulation and/or hemoclips.

### 3.3. Comparison among the different Forrest classifications

No significant differences in the age and sex of the patients were found with different the Forrest classifications (P > 0.05, Table 3). The comparison of the GBS/SI/rebleeding rates among the different Forrest classifications showed statistical significance (P < 0.05). Both the GBS and SI showed a peak value in patients with Forrest IIa, and the GBS was significantly higher in patients with Forrest IIa than in those with Ib/IIc/III (P < 0.05), while SI was significantly higher in Forrest IIa than Ib/IIb/III (P < 0.05). There was no statistically significant difference in the GBS**/**SI among the other Forrest classifications (P > 0.05). The total rebleeding rate was 4.08%; the rebleeding rate in Forrest Ia (20.00%) was higher than in Forrest III (1.67%), with statistical significance (P < 0.0033); the rebleeding rate in Forrest Ib (3.70%) was lower than in Forrest IIa (21.59%), with statistical significance (P < 0.0033); and the rebleeding rate in Forrest IIa was higher than in Forrest IIb/IIc/III, also with statistical significance (P < 0.0033) (Table 3). 

**Table 3:** Comparison among different Forrest classifications.

Variable	Ia (n = 15)	Ib (n = 81)	IIa (n = 88)	IIb (n = 76)	IIc (n = 36)	III (n = 659)	F/c2	P
Age (years), mean ± SD	63.27 ± 13.13	54.23 ± 17.04	58.98 ± 13.26	57.25 ± 15.95	57.64 ± 15.45	57.10 ± 15.01	1.362	0.236
Sex (male/female)	13/2	64/17	73/15	55/21	27/9	469/190	8.544	0.129
GBS, mean ± SD	9.80 ± 3.26	9.40 ± 3.18a	10.58 ± 3.39cd	9.66 ± 3.36	8.75 ± 3.64	8.95 ± 3.34	4.315	0.001
SI, mean ± SD	0.81 ± 0.18	0.79 ± 0.21a	0.85 ± 0.19bd	0.79 ± 0.21	0.79 ± 0.17	0.75 ± 0.18	5.686	<0.001
Rebleeding, n (%)	3 (20.00)	3 (3.70)	19 (21.59)	3 (3.95)	0 (0.00)	11 (1.67)	89.935	<0.001*

a Compared with Forrest IIa, P < 0.05; b compared with Forrest IIb, P < 0.05; c compared with Forrest IIc, P < 0.05; d compared with Forrest III, P < 0.05; *P < 0.05, the test level α’ of the pairwise comparison ratio was = 0.0033 (P: subgroup Ia vs. subgroup III: 0.003, subgroup Ib vs. subgroup IIa: 0.001, subgroup IIa vs. subgroup IIb: 0.001, subgroup IIa vs. subgroup IIc: 0.002, and subgroup IIa vs. subgroup III: <0.001);

### 3.4. Correlation analysis between the GBS and SI

**Figure 2:**
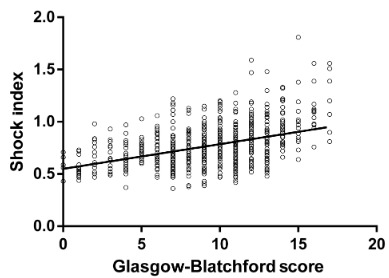
Glasgow-Blatchford score-shock index scattering dots curve.

The GBS-SI scattering dot curves showed clustering using the curve imitation method. Pearson’s analysis revealed that the GBS was positively correlated with the SI (P < 0.001), at r = 0.427 (Figure 2). 

### 3.5. Correlation analysis between the GBS /SI and the Forrest classification

Spearman’s rank analysis revealed a negative correlation between the GBS and Forrest classification, at r = –0.111 (P < 0.01), and between the SI and Forrest classification, at r = –0.138 (P < 0.01).

## 4. Discussion

According to reports, the incidence of PUB ranges from 20 to 60/100,000 people and the mortality rate remains at 5%–10%, despite advances in endoscopy and medication [13]. Early assessment of the disease severity and prognosis has become increasingly important. In this study, it was determined that the GBS in patients with PUB was positively correlated with the SI, the correlation between the Forrest classification and the GBS or SI was relatively low; the GBS, SI, and the rebleeding rates in patients with Forrest Ib were significantly lower than in those with Forrest IIa.

The application value of the GBS in assessing the condition of a patient, need for intervention, and evaluation of the prognosis for patients with upper gastrointestinal bleeding has been confirmed by numerous studies [14–16]. The European Society of Gastrointestinal Endoscopy has recommended assessment using the GBS before endoscopy, with low-risk (GBS 0–1) patients not requiring early endoscopy or hospitalization [17]. Some studies have found that the GBS can better predict rebleeding in patients with upper gastrointestinal bleeding [18–20], and a high GBS (GBS > 7) is associated with the risk of rebleeding [21]. 

The SI can provide a comprehensive assessment of cardiovascular status and can be used to estimate the amount of blood loss and degree of shock (normal range: 0.5 to 0.7) [7]. A study by Rassameehiran et al. showed that the SI was a good tool to identify patients with the potential for short-term adverse outcomes when they presented with upper gastrointestinal bleeding, and the SI was performed, as well as other risk-scoring tools, for gastrointestinal bleeding [22]. In this study, it was determined that the GBSs of patients with PUB were positively correlated with the SI, i.e., the higher the SI, the higher the GBS. Clinically, the SI can be more easily calculated than the GBS. Therefore, emergency patients should first be assessed with the SI to determine their disease severity, and the GBS may be used to further assess their disease severity and prognosis after the completion of blood and other tests, as it may be helpful for risk stratification and decision-making clinically before endoscopy. 

With the wider application and higher importance of emergency endoscopy, identifying the cause of bleeding as early as possible via endoscopy is of great significance for the diagnosis and treatment in ANVUGIB patients [8,23]. The Forrest classification is an endoscopic scoring system, which classifies ulcer lesions into high-risk and low-risk, and it is helpful in predicting the risk of rebleeding and guiding endoscopic hemostasis therapy [17]. At present, the analysis of risk factors on adverse outcomes of ANVUGIB patients [24] and the comparison of the GBS with other risk scores (e.g., Rockall score, AIMS65, etc.) in predicting the prognosis of ANVUGIB patients are still hot topics, and some other researchers have reported the application value of the GBS and Forrest classification in Mallory-Weiss syndrome [25]. However, the correlation between the clinical severity score (such as the GBS and SI) and the severity of endoscopic manifestations (Forrest classification) in patients with PUB has not been reported.

The study herein explored the correlation between the GBS or SI before endoscopy and the Forrest classification after endoscopy, and it was found that the correlation between the Forrest classification and GBS or SI was relatively low. The r values were only –0.111 and –0.138, respectively, with several possible explanations, as follows: 1) Due to the small sample size, with only 15 patients (1.57%) with Forrest class Ia, we could not perform adequate investigation on those with a high rebleeding risk; thus, a larger sample and further research are needed. 2) PPIs are important in the treatment of PUB. Moreover, preendoscopy PPI treatment can improve the condition of a bleeding peptic ulcer and reduce the need for endoscopic treatment [26,27]. All of the patients in the current study used PPIs prior to gastroscopy, possibly reducing the Forrest grade of the patients. 3) As heart rates and blood pressures of the patients changed dynamically, performing dynamic monitoring to determine the SI may be preferred.

In addition, the study showed that Forrest Ib patients had lower a GBS and SI than those with Forrest IIa before endoscopic examination, the rebleeding rate in the Forrest Ib patients was lower than in those with Forrest IIa after initial treatment (including endoscopic hemostasis), and the rebleeding rate in patients with Forrest Ib (3.70%) was indeed low after initial treatment, which was consistent with recent findings [28], but the value of the Forrest Ib classification as a sign of high-risk ulcers may need to be reevaluated.

There were several limitations in this study: 1) This was a retrospective study, and the sample was small; thus, a larger sample and prospective study are needed to confirm the results. 2) *H. pylori* infection is the main cause of peptic ulcer. The eradication of *H. pylori* is closely related with the prognosis of patients with peptic ulcer, e.g., rebleeding, and it can promote ulcer healing. In this study, 427 patients (44.71%) tested positive for *H. pylori* and were treated with the standard *H. pylori* eradication therapy, but the efficacy of the therapy was not followed up. At the same time, patients who had tested negative for *H. pylori* infection during bleeding were not rechecked, and some patients did not have any test results for *H. pylori*. Hence, future studies should pay close attention to *H. pylori* eradication therapy as well as its effect on the prognosis of the patient. 3) Different endoscopists and methods used for endoscopic hemostasis may have influenced the rebleeding rate. 4) The study did not compare the comorbidities of the different Forrest classifications, and as comorbidities are risk factors for rebleeding, further studies are needed to analyze the rebleeding rates and risk factors corresponding to the Forrest classifications. 5) The incidence of rebleeding was only recorded for 7 days after the initial treatment; hence, longer-term follow-up of the rebleeding rate, e.g., over 30 days, may be necessary.

In conclusion, the moderate correlation between the GBS and SI may be helpful for risk stratification and clinical decision making before endoscopy. Correlation between the Forrest classification and the GBS or SI was relatively low. 

## Acknowledgments

This work was funded by the Agency Issued Joint Fund of Guizhou Province Science and Technology Department of Guiyang Medical University ([2010]3169).

## Informed consent

This study was conducted in accordance with the declaration of Helsinki. It was approved by the Medical Ethical Committee of the Affiliated Hospital of Guizhou Medical University. Written informed consent was obtained from all of the patients prior to the endoscopic procedures.

## References

[ref1] (2013). Acute upper gastrointestinal bleeding (UGIB)-initial evaluation and management. Best Practice and Research Clinical Gastroenterology.

[ref2] (2015). Acute, nonvariceal upper gastrointestinal bleeding. Current Opinion in Critical Care.

[ref3] (2000). A risk score to predict need for treatment for upper-gastrointestinal haemorrhage. Lancet.

[ref4] (2009). Outpatient management of patients with low-risk upper-gastrointestinal haemorrhage: multicentre validation and prospective evaluation. Lancet.

[ref5] (1995). Risk assessment scores for patients with upper gastrointestinal bleeding and their use in clinical practice. Hospital Practice.

[ref6] (2015). Performance of new thresholds of the Glasgow Blatchford score in managing patients with upper gastrointestinal bleeding. Clinical Gastroenterology and Hepatology.

[ref7] (2016). Utility of the shock index and other risk-scoring tools in patients with gastrointestinal bleeding. Southern Medical Journal.

[ref8] (2014). Endoscopy for nonvariceal upper gastrointestinal bleeding. Clinical Endoscopy.

[ref9] (2008). Management of acute bleeding from a peptic ulcer. The New England Journal of Medicine.

[ref10] (2015). Endoscopic management of peptic ulcer bleeding. Clinical Endoscopy.

[ref11] (2014). Reassessment of the predictive value of the Forrest classification for peptic ulcer rebleeding and mortality: can classification be simplified. Endoscopy.

[ref12] (2007). The Strengthening the Reporting of Observational Studies in Epidemiology (STROBE) statement: guidelines for reporting observational studies. Annals of Internal Medicine.

[ref13] (2010). Causes of mortality in patients with peptic ulcer bleeding: a prospective cohort study of 10,428 cases. American Journal of Gastroenterology.

[ref14] (2018). Asia-Pacific working group consensus on non-variceal upper gastrointestinal bleeding: an update 2018. Gut.

[ref15] (2014). Is the Glasgow Blatchford score useful in the risk assessment of patients presenting with variceal haemorrhage?. European Journal of Gastroenterology & Hepatology.

[ref16] (2018). Non-variceal upper gastrointestinal bleeding. Nature Reviews Disease Primers.

[ref17] (2015). Diagnosis and management of nonvariceal upper gastrointestinal hemorrhage: European Society of Gastrointestinal Endoscopy (ESGE) Guideline. Endoscopy.

[ref18] (2016). Comparison of AIMS65, Glasgow-Blatchford score, and Rockall score in a European series of patients with upper gastrointestinal bleeding: performance when predicting in-hospital and delayed mortality. United European Gastroenterology Journal.

[ref19] (2016). Comparison of Glasgow-Blatchford score and full Rockall score systems to predict clinical outcomes in patients with upper gastrointestinal bleeding. Clinical and Experimental Gastroenterology.

[ref20] (2017). Scoring systems for peptic ulcer bleeding: Which one to use?. World Journal of Gastroenterology.

[ref21] (2016). High Glasgow Blatchford score at admission is associated with recurrent bleeding after discharge for patients hospitalized with upper gastrointestinal bleeding. Endoscopy.

[ref22] (2017). Utility of the shock index for risk stratification in patients with acute upper gastrointestinal bleeding. Southern Medical Journal.

[ref23] (2012). endoscopy in elderly patients with non-variceal upper gastrointestinal bleeding. Videosurgery and Other Miniinvasive Techniques.

[ref24] (2019). Epidemiology and risk factors of adverse outcome in nonvariceal upper gastrointestinal bleeding. Khirurgiia (Mosk).

[ref25] (2016). Effective endoscopic treatment of Mallory-Weiss syndrome using Glasgow-Blatchford score and Forrest classification. Journal of Digestive Diseases.

[ref26] (2010). International consensus recommendations on the management of patients with nonvariceal upper gastrointestinal bleeding. Annals of Internal Medicine.

[ref27] (2014). Are proton-pump inhibitors effective treatment for acute undifferentiated upper gastrointestinal bleeding?. Annals of Emergency Medicine.

